# Supplementary material for: Age-Dependent Surface Receptor Expression Patterns in Immature Versus Mature Platelets in Mouse Models of Regenerative Thrombocytopenia
Source: Cells. 2023 Oct 8;12(19):2419. doi: 10.3390/cells12192419 (PMC10571991; doi:10.3390/cells12192419)
Supplement: Supplementary file 1 [file cells-12-02419-s001.zip › cells-2641371-supplementary.pdf]

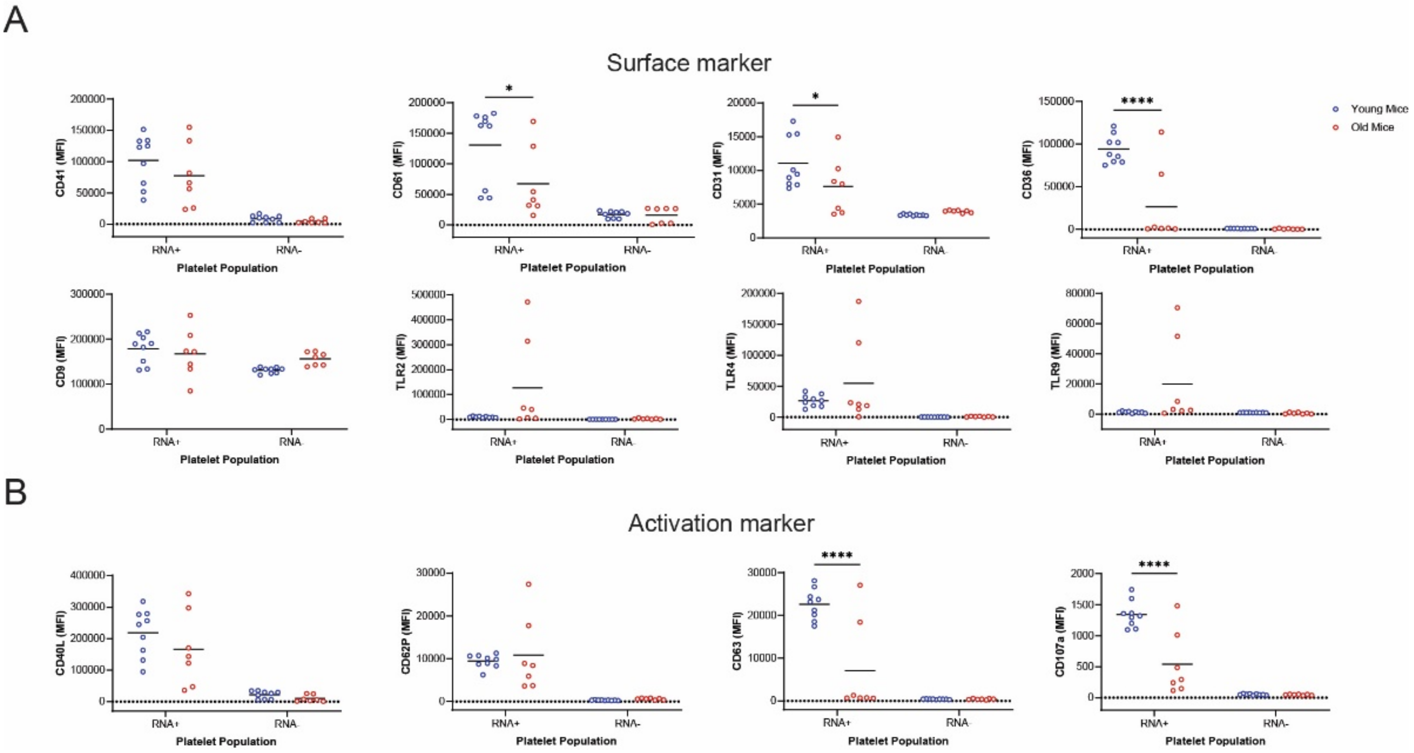

Figure S1. Quantification of surface receptor and activation markers on immature and mature platelets in young and old mice: (**A**) and (**B**) PRP was stained with Syto RNASelect to identify immature platelets and antibodies to quantify distinct surface (**A**) and activation markers (**B**) were analysed by flow cytometry. Scatter diagrams compare receptor expression on immature (RNA+) and mature (RNA-) platelets between young (10-weeks) and old (50-weeks) mice, respectively. The measure of central tendency denotes the mean (**A** and **B**). Different *p*-values $p < 0.05$, and $p < 0.0001$ are indicated as *, and ****, respectively.

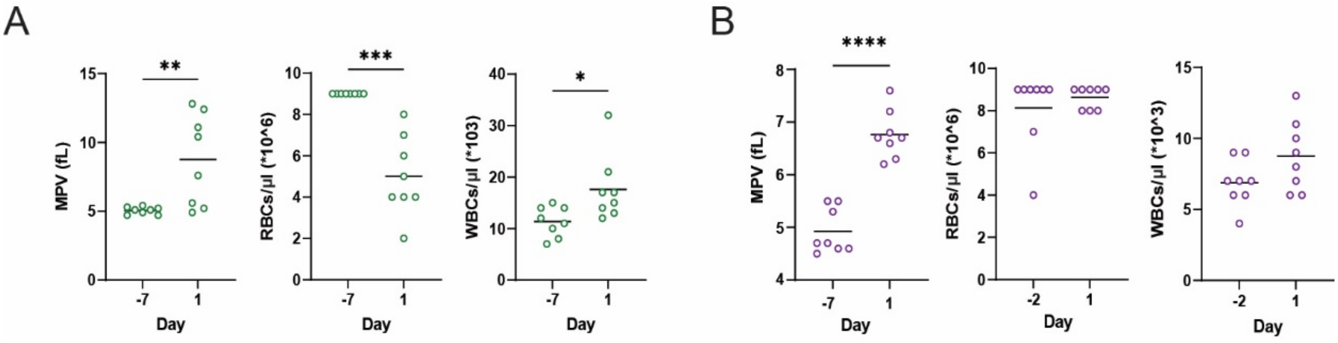

Figure S2. Hematological parameters of iDTR and R300-mediated mouse platelet depletion models on the first day of regenerative thrombocytopenia: (**A**) and (**B**) Blood was drawn before initiation of platelet depletion and one day after platelet nadir and analysed with an automated hematology analyzer. Scatter charts visualise the mean platelet volume (MPV), number of red blood cells (RBCs) and white blood cells (WBCs) of (**A**) iDTR and (**B**) R300 mouse models. Different *p*-values $p < 0.05$, $p < 0.01$, $p < 0.001$, and $p < 0.0001$ are indicated as *, **, ***, and ****, respectively.

Table S1. List of anti-mouse antibodies used for flow cytometry analyses and immunohistochemistry.

Antibody/Dye	Source	Concentration	Clone
Anti-CD9 eF450	Invitrogen	1μg/mL	eBioKMC8 (KMC8)
Anti-CD31 eF450	Invitrogen	1μg/mL	390
Anti-CD36 PerCP/Cy5.5	BioLegend	1μg/mL	HM36
Anti-CD41 AF488	BioLegend	10μg/mL	MWReg30
Anti-CD41 AF750	R&D Systems	10μg/mL	386629
anti-CD61 BV650	BD Biosciences	4μg/mL	2C9.G2
Anti-CD62P PE/Cy7	BioLegend	1μg/mL	RMP-1
Anti-CD63 APC-Cy7	BioLegend	1μg/mL	NVG-2
Anti-CD107a BV510	BioLegend	2μg/mL	1D4B
Anti-CD145 APC (CD40L)	BioLegend	8μg/mL	MR1
Anti-CD284 APC (TLR2)	BioLegend	1μg/mL	QA16A01
Anti-CD284 PE/Cy7 (TLR4)	BioLegend	1.33μg/mL	MTS510
Anti-CD289 AF405 (TLR9)	R&D Systems	1μg/mL	1138D
Hoechst33342	Invitrogen	5μg/mL	
Syto RNASelect green fluorescent cell stain	Invitrogen	1μM	
